# [Retracted] miR‑187 inhibits the growth of cervical cancer cells by targeting FGF9

**DOI:** 10.3892/or.2023.8632

**Published:** 2023-09-18

**Authors:** Hua Liang, Ruoyu Luo, Xiaoqi Chen, Yuzi Zhao, Aili Tan

Oncol Rep 38: 1977–1984, 2017; DOI: 10.3892/or.2017.5916

Following the publication of this paper, it was drawn to the Editor's attention by a concerned reader that the cell formation assay data shown in Figs. 3C, 4C and 7C, and certain of the western blotting data shown in Figs. 7E and 8E were strikingly similar to data that had already appeared in other articles written by different authors at different research institutes. Owing to the fact that the contentious data in the above article had already been published prior to its submission to *Oncology Reports*, the Editor has decided that this paper should be retracted from the Journal. The authors were asked for an explanation to account for these concerns, but the Editorial Office did not receive a reply. The Editor apologizes to the readership for any inconvenience caused.

